# Evaluation of home-based naturopathic telehealth clinic: an innovative COVID-19 pandemic response

**DOI:** 10.1186/s13104-022-06140-x

**Published:** 2022-08-01

**Authors:** Tracelee Shew, Catherine Smith, Greg Connolly, Craig S. McLachlan

**Affiliations:** 1 Health, Torrens University Australia, 17-51 Foveaux St, Surry Hills, Sydney, 2010 Australia; 2 Health, Torrens University Australia, 196 Flinders St, Melbourne, 3000 Australia; 3 Centre for Healthy Futures, Torrens University Australia, 17-51 Foveaux St, Surry Hills, Sydney, 2010 Australia

**Keywords:** Telehealth, Naturopathy, Clinical Practicum, Collaborative, Peer learning, Education

## Abstract

**Objectives:**

The COVID-19 pandemic in Australia disrupted usual clinical training placements for naturopathic students. An innovative, remote Telehealth clinic was developed and implemented. This pilot study evaluates student and educator learning and teaching experiences in Telehealth. A survey assessed Likert and qualitative written responses to student and staff interaction with the Telehealth clinic.

**Results:**

Nine student and 12 educator responses were included in the analysis. All students positively rated Telehealth training resources and the educator support provided. Students rated the Telehealth learning experience as ‘very good’ (78%) or ‘good’ (22%) with educator ratings of ‘very good’ (67%) or ‘good’ (33%). Thematic analysis of student written responses showed increased client diversity, collaboration, peer learning, increased feedback, and improved digital and technology skills. Virtual physical examination and infrastructure limitations were reported as Telehealth clinical practicum challenges.

Naturopathic Telehealth clinic practicum is a valuable alternative to in-person clinical practicums for Australian students. It enhances student collaboration and peer learning. Challenges of technology, infrastructure and incorporating Telehealth in curriculum may be barriers to implementation of Telehealth. However, Telehealth is an important clinical training option to prepare student practitioners for contemporary professional practice if in-person consultation is prohibitive, such as during the COVID-19 pandemic.

**Supplementary Information:**

The online version contains supplementary material available at 10.1186/s13104-022-06140-x.

## Introduction

Naturopathy is a system of health care based on traditional philosophies that utilise a wide variety of techniques to achieve improved health outcomes [1]. In Australia, Naturopathy is a self-regulated profession where clients access Naturopathic care to complement medical or allied health care and to proactively prevent or treat acute and chronic conditions [2].

Pre-COVID-19 research indicates that 60% of Australian complementary medicine practitioners offer online or telephone consultations in addition to in-person consultations [2]. Despite complementary medicine practitioners using technology-enabled consultations, Telehealth was not included in the naturopathic educational curriculum. The COVID-19 pandemic caused significant disruption to in-person clinic training placements for students [3, 4]. Naturopathic educators faced the challenge of developing a home-based clinic experience that mimicked the in-person consultations and provided students with the opportunity to consult online with clients under supervision. Australian educators were guided by the Tertiary Education Quality and Standards Agency (TEQSA) guidelines detailing reasonable adjustments to clinical practicum during the pandemic, and professional association clinic requirements [5, 6]_._ To achieve this outcome, an innovative remote Telehealth clinical service was developed and implemented.

To the authors’ knowledge, there is limited published literature relating to Naturopathic students and educators' experiences of using remote Telehealth clinics. Previous research reported complementary medicine students are more likely to use Telehealth in future clinical practice than educators [7, 8]_._ There is emerging research evaluating home-based Telehealth consultations implemented during the COVID-19 pandemic in medicine [9, 10] and dietetics [11]. This study aims to describe the initial student-led remote Telehealth clinic implemented during the COVID-19 pandemic and to evaluate student and supervisor learning and teaching experience. An electronic survey was completed by students and educators who participated in the 2020 clinic, sent via university email.

## Main text

### Methods

#### Telehealth

Videoconferencing was selected as the consultation technology for telehealth. Microsoft Office 365 and Blackboard Collaborate were existing platforms available to the university staff and students. Digital infrastructure was designed to meet data security, home-based confidential access, availability of technology and ease of use.

#### Design of the telehealth clinical practicum

Groups of up to six students and one clinical supervisor were allocated to each clinical practicum session of 6 hour duration for a 12-week trimester. To support students and educators’ transition to Telehealth, a National Telehealth Training Program and curriculum documents were developed. Virtual training sessions were provided to all educators and students. The Academic Clinic Leaders and technical specialists were available for support.

#### Study design

Two surveys, one for educators and one for students, were developed by the research team and were based on the University academics clinical expertise (Additional file 1: Table S1, S2). The surveys investigated Telehealth implementation and delivery in a national student health clinic program. These provided insights into satisfaction with Telehealth, implementation issues, program quality, and seeking recommendations for further supportive needs in technical and clinical training support.

Electronic surveys were sent to participants university email by a data collection researcher and completed online. A follow-up reminder email was sent two weeks later. The study was approved by The Torrens University Australia Human Research Ethics Committee (Project ID 74) to audit the pilot Telehealth clinic data set. All participants were informed about specifications of the study, including an ability to opt-out of the evaluation, and signed an informed consent agreement.

The surveys contained quantitative and qualitative questions designed to gain evaluative and descriptive feedback on the Telehealth learning and teaching experience. A 5-point Likert scale was used regarding questions on support and professional development, with students rating Telehealth training and resources provided. A “yes” or “no” was used on student future use of telehealth clinic. Written questions provided further feedback on advantages, disadvantages and improvements for telehealth.

#### Participants

Adult university students, of both sexes, enrolled in a clinical practicum subject of the Bachelor of Health Science degree, and educators employed to supervise students during the clinical practicum, were initially recruited. A total of 49 students and 12 supervisors, participated in the Telehealth clinic, of these 46 completed the survey and 21 participants returned the Informed consent agreement, students (*n* = 9) and educators (*n* = 12) across campuses.

The exclusion criteria included observing preclinical students and clinical administration staff. While the initial survey responses were not blinded to the researchers, the data collection researcher removed respondent names, identifying data and comments prior to data set audit.

#### Data analysis

Qualitative data was thematically analysed using a scoring system for similarly themed responses [12].

## Results

### Training, resources and support

Students rated the telehealth training, learning experience and resources provided to them as “very good” (78%) and “good” (22%). The educators’ perspective on student learning experience in telehealth were slightly lower with “very good” (67%) and “good"(33%).

All students felt “extremely” supported during Telehealth clinic (100%). Confidence implementing telehealth into student practitioners own future practice, responses were “extremely” confident (78%) and “somewhat” (22%). 89% of students rated the value of learning telehealth for professional development, as “extremely” (89%) valuable, with 11% choosing “somewhat”. In total, 87% of participants responded “yes” and 11% “maybe” regarding the inclusion of telehealth for future university clinic courses.

### Telehealth advantages

All participants completed qualitative written responses with examples of the responses included in Additional file 2: Table S3. The advantages of telehealth include the ability to increase client diversity, more detailed feedback from educators and better collaboration with peers (Table 1). The smaller groups were reported as being positive learning experiences by all participants. Students reported improved digital skills and technology capability and educators stated students developed improved confidence and competence in Telehealth during the trimester.

**Table 1:** Student Practitioner feedback—Telehealth advantages

Themes	Responses (%)
Increased client diversity	24
Increased supervisor feedback	20
Increased peer learning and collaboration	16
Improved digital consult, technology skills	16
More preparation time, reduced travel	8
Increased ability to source clients	8
Improved client comfort, waiting time	4
Improved case taking and analysis skills	4

### Telehealth disadvantages

Disadvantages of telehealth were noted in the qualitative written responses. Students and educators reported (Table 2) the inability to conduct comprehensive physical examinations. Educators and students (Table 3) were initially challenged using Telehealth technology including E-digital records management. Reduced capacity for client appointments due to smaller clinic sizes, and a lack of in-person dispensing experience, were reported by both students and educators. Students also commented on having to complete additional in-person clinical hours due to Australian Professional Associations requirements.

**Table 2:** Student Practitioner feedback—Telehealth disadvantages

Themes	Responses (%)
Inability for physical examination	22
Decreased client appointments	17
Not able to dispense in-person	12
Limitation on body language cues	12
Technology not always reliable	11
Additional in-person hours required	11
Decreased ability for client rapport	5
Increased digital record management	5
Increased student performance anxiety	5

**Table 3:** Thematic analysis of Educator responses

Themes	Responses (%)
Positive learning experiences (*n* = 54)	
Small group learning environment	20
Increased time with supervisor	18
Student observation of entire consultation process	18
Increased student collaboration and peer support	15
Increased student confidence & competence in telehealth	13
Increased client diversity	12
Perceived decrease in student stress levels	4
Challenges (*n* = 34)	
Technical issues	24
Lack of ability to conduct in-person physical examination	18
Remote practice administration	15
Limitations on client appointment numbers	15
Digital records management	15
Managing student performance anxiety	5
Assessing professional skills	3
Managing student external dispensary product knowledge	3
Impersonal learning environment	2

## Discussion

### Educational value

This study is the first to evaluate the learning and teaching experience of a remote naturopathic student-led Telehealth clinic that was made operational due to COVID-19. A search of the literature sourced three studies that also evaluated home-based Telehealth teaching clinics [9–11] and two novel Telehealth on-consultation training models [13, 14]. All studies reported high educational value and positive student learning outcomes.

### Diversity of clients

Students reported that the Telehealth clinic attracted an increased diversity of clients compared to in-person clinic. Similar outcomes are reflected in an Australian remote Dietetics clinic where students conducted telephone or video-consultations [11]. The researchers suggest that removing consultation cost and travel barriers contributed to the success of the Telehealth clinic [11]. The medical remote e-consult models with live client consultations are limited but have been reported as feasible and successful [9, 10, 13, 14]. Students reported that Telehealth improved clinical reasoning and level of comfort with clinical decisions [10].

### Clinical Learning

The remote Telehealth clinic was unique in providing multiple online platforms to enable consultations as well as case discussions. This allowed for peer observation of skills, immediate and direct supervisor feedback on performance and small group collaborative peer learning. Synchronous pre-brief and de-brief discussions are a differentiating factor compared to remote medical e-consult models that did not have a pre-brief opportunity [10] and sometimes required students and supervising clinicians to debrief later [9]. When a remote student joins an in-patient e-consult, students reported a positive aspect of the learning experience was to work closely with the supervisor in a one-to-one teaching environment. This contrasts with educator feedback that recommended building larger group collaborations to encourage team-based learning [9].

Our study identified that near-peer student observation of the entire consultation process was regarded as a positive learning experience. Similarly, in 2021 Darnton et al*.* [10] reported that observing students were more engaged in Telehealth due to the possibility that they could be called upon to step in as the primary practitioner if a technical malfunction occurred. Also in 2021, Manjunatha et al. [13] identified that direct skill transfer was a learning advantage of a novel on-consultation study where primary care doctors live-steamed real-time telepsychiatry consultations to observing students. In a similar remote neurology clerkship, students observed real-patient Telehealth appointments conducted by the physician and debriefed with the teaching facility following the appointment [14]. These studies confirm the findings of this Naturopathic Telehealth research that Telehealth facilitates important clinical skills development through peer observation.

### Telehealth training

One strength of the Naturopathic Telehealth clinic is the comprehensive Telehealth training program and in-session technical support. A similar method of training is described in a 2021 study by Frankl and colleagues where 252 medical students at Harvard Medical School engaged in a 5-module curriculum. The results show students self-rated Telehealth knowledge statistically higher (p < 0.001) following the course [15]. Students reported asynchronous learning materials to be a positive learning experience.

Regarding Telehealth curricula, a scoping systematic review concluded that Telehealth curricula in allied health programs is limited [16]. Similarly in biomedicine, implementation of Telehealth curricula into undergraduate programs is highly recommended [17]. As there is no current discourse on Telehealth competencies in naturopathic education there is an urgent need for formal curricula development.

### Telehealth challenges

Inability of students to complete physical examination is identified as a disadvantage of Telehealth in studies [9, 10]. However, third and fourth year medical residents participating in an outpatient teleneurology clinic for 4 weeks, found that video consultations were the same or superior to in-person visits for obtaining a comprehensive case history, formulating treatment plans and communicating recommendations [18]. A qualitative study by Donaghy et al. of patient and clinician video-conference experience identified that keen observation by clinicians of visual cues was a positive communication aid and developing client-centred Telehealth communication competencies is essential for best practice virtual care [19]. Conversely, Donaghy et al. [19] stated that for complex problems, in-person consultation is preferred. However, a review by Alkureishi et al. [20] argues that Telehealth can be used in most circumstances and educators can train students to identify which cases are appropriate for online physical examinations.

Despite several technical issues, the digital and technology skills and confidence of students and educators improved during the Telehealth experience. Similar research suggests it is important to establish processes for Telehealth especially trouble-shooting internet disruption issues [10, 11]. Many articles in medical literature explore the benefits of pre-clinical Telehealth training and objective structured clinical examinations to better prepare students for live Telehealth placements [15–17, 21, 22]. This is an area for future development in the naturopathic curricula.

A 2017 cross sectional survey reported on two complementary medicine education institutions in Australia and USA. The pre-Covid-19 study found 43% of students were interested in using Telehealth, 49% of students were hesitant, and only 15.9% of educators used Telehealth [7, 8]. Our study is unique as home-based students and educators were able to consult with live clients during the pandemic. Whilst advantages and disadvantages were identified, students and educators recognise the importance of Telehealth to future-proof practice. This sentiment is reflected in the medical literature with institutions recognising the value of permanent Telehealth training and clinical experience [14, 16, 23].

## Limitations

This was a multicentred, single university pilot study. The small sample size of participants in this survey study precludes generalisability of these results in other Telehealth contexts. Challenges exist when accessing a small sample size, with an inability to reach statistical significance. Future studies could add methodological improvements by utilising mixed methods with interviews, focus groups, observation, and participatory action methods, that take place with larger participant groups across multi-centred settings. The issues explored in this study may be transferable to future, larger studies in this important, nascent development of clinical training and client care via Telehealth. This study adds to the small body of knowledge in this field that points to the positive benefits of student-led Telehealth consultations.

## Supplementary Information

**Additional file 1: Table S1**. Students investigated their experiences of telehealth implementation and delivery in clinical care. **Table S2**. Educators examined their insights into student learning experiences and competencies with Telehealth.

**Additional file 2: Table S3**. Qualitative response examples from participants.

## Data Availability

The datasets used and/or analysed during the current study are available from the corresponding author on reasonable request.
