# Supplementary material for: Evaluation of home-based naturopathic telehealth clinic: an innovative COVID-19 pandemic response
Source: BMC Res Notes. 2022 Aug 1;15:269. doi: 10.1186/s13104-022-06140-x (PMC9342589; doi:10.1186/s13104-022-06140-x)
Supplement: Supplementary file 1 — Additional file 1: Table S1. Students investigated their experiences of telehealth implementation and delivery in clinical care. Table S2. Educators examined their insights into student learning experiences and competencies with Telehealth. [file 13104_2022_6140_MOESM1_ESM.docx]

Additional file 1 of the Telehealth Student and Educator Surveys

Table S1: Telehealth Student Survey

1. First Name

Written answer

1. Last Name

Written answer

1. What qualification are you studying?
   1. Bachelor of Health Science (Naturopathy)
   2. Bachelor of Health Science (Western Herbal Medicine)
2. Campus Location
   1. Brisbane
   2. Sydney
   3. Melbourne
3. Indicate which clinical practicum/s you attended during the Telehealth clinics.
   1. CAM401A or CAM309A
   2. CAM403A or CAM310A
   3. CAM404A or CAM316A
   4. CAM408A or CAM317A
   5. CAM409A or CAM318A
4. How would you rate the Telehealth Training and Resources provided?
   1. Very good
   2. Good
   3. Neutral
   4. Poor
   5. Very Poor
5. For each clinic session over the trimester, how many times did you act as the Primary Practitioner?
   1. 5 or more
   2. 4
   3. 3
   4. 1-2
   5. 0
6. For each clinic session over the trimester, how many times did you observe or act as the Secondary Practitioner?
   1. 15 or more
   2. 10 or more
   3. Between 5 and 9
   4. Between 1 and 4
7. How would you rate your learning experience in the Telehealth format?
   1. Very good
   2. Good
   3. Fair
   4. Poor
   5. Very Poor
8. List five (5) advantages of learning in the Telehealth Clinic?

Written answer

1. List five (5) disadvantages of learning in the Telehealth Clinic?

Written answer

1. How supported did you feel in the Telehealth Clinic?
   1. Extremely supported
   2. Somewhat supported
   3. Neutral
   4. Somewhat not supported
   5. Extremely not supported
2. Having experienced a Telehealth Clinic, how confident do you feel implementing Telehealth in your future practice?
   1. Extremely confident
   2. Somewhat confident
   3. Neutral
   4. Somewhat not confident
   5. Extremely not confident
3. How valuable has learning in a Telehealth environment been to your professional development?
   1. Extremely valuable
   2. Somewhat valuable
   3. Neutral
   4. Somewhat not valuable
   5. Extremely not valuable
4. What improvements can we make to the Telehealth Learning Experience?

Written answer

1. Would you recommend Telehealth be included as part of the Clinical Practicum learning experience in the future?
   1. Yes
   2. No
   3. Maybe
2. Any other comments?

Written answer

END OF SURVEY

Table S2. Telehealth Educator Survey

1. First Name

Written answer

1. Last Name

Written answer

1. Campus Location
   1. Brisbane
   2. Sydney
   3. Melbourne
2. How would you rate the student learning experience in the Telehealth format?
   1. Very good
   2. Good
   3. Neutral
   4. Poor
   5. Very Poor
3. Do you have any students who have had less than 5 x Primary Practitioner case taking experiences?
   1. Yes
   2. No
   3. Maybe
4. Do you have any students who have not yet demonstrated adequate clinical competence?
   1. Yes
   2. No
   3. Maybe
5. What student learning experiences have been challenging in the Telehealth clinic?

Written answer

1. What student learning experiences have been positive in the Telehealth clinic?

Written answer

1. What improvements can we make to the Telehealth Learning Experience?

Written answer

1. Any other comments?

Written answer

END OF SURVEY
