# Supplementary material for: Evaluation of home-based naturopathic telehealth clinic: an innovative COVID-19 pandemic response
Source: BMC Res Notes. 2022 Aug 1;15:269. doi: 10.1186/s13104-022-06140-x (PMC9342589; doi:10.1186/s13104-022-06140-x)
Supplement: Supplementary file 2 — Additional file 2: Table S3. Qualitative response examples from participants. [file 13104_2022_6140_MOESM2_ESM.docx]

Additional file 2 of the Qualitative response examples from participants

Table S3: Qualitative response examples from participants

| Physical Assessment | “*Unable to do proper physical assessments on clients”*  *“Probably the most important component that the online platform cannot provide is the physical examinations”*  *“The only concern I have is around practising physicals”* |
| --- | --- |
| Client Diversity | "..*.good exposure to a range of interesting telehealth clients that would not have been able to attend in person*”  “*Continuing to have clients and perhaps attracting more clients that would otherwise be able to attend clinic*” |
| Technology | *“Initially at the beginning of T2 the biggest challenge for students was getting familiar with the TEAMS platform”*  *“Disadvantages were mainly based around the complications of learning to deal with the technology and how it can let you down, e.g., poor internet signal for client, signals dropping out”*  *“Tech and connection issues presented a challenge in first few weeks of T2 - on all sides - student, client, platform, e-forms”*  *“Some of the tech issues (like patient dropping out etc), are realistic of what can happen in practice”* |
| Class size | *“Small class numbers allowed for more focused tuition and assessment”*  *"... small groups so the supervisors get to see all consults and great group discussions”*  *“All of them have mentioned numerous times how valuable the smaller group experience was”* |
| Clinics Online | *“Should still be offered even in F2F clinic, as some patients (returns specifically) would be more than likely re-book"*  *“Once students developed confidence with the online platform, they showed excellent learning ability”*  *“Some students enjoy face to face consults more than online”*  *“Students have now become familiar with a Telehealth platform and their tele-health skills are becoming polished!”* |
| Currency of Telehealth | *“The benefits of including it as part of student clinical practicum learning is great practice before going out into the workforce “*  *“Even without the current pandemic, we are studying in 2020 and the world is moving away from face-to-face to online; learning how to safely so telehealth is a must”*  *“Working from home, less travel time, good experience in the Telehealth platform for future consultation possibilities”* |
